# (Smoldering) multiple myeloma: mismatch between tumor load estimated from bone marrow biopsy at iliac crest and tumor load shown by MRI

**DOI:** 10.1007/s00256-023-04383-8

**Published:** 2023-06-10

**Authors:** Fabian Bauer, Sandra Sauer, Niels Weinhold, Stefan Delorme, Markus Wennmann

**Affiliations:** 1 Division of Radiology, German Cancer Research Center (DKFZ), Im Neuenheimer Feld 280, 69120 Heidelberg, Germany; 2 Department of Medicine V, Multiple Myeloma Section, University Hospital Heidelberg, Im Neuenheimer Feld 410, 69120 Heidelberg, Germany

**Keywords:** Magnetic resonance imaging, Multiple myeloma, Monoclonal plasma cell disorders, Tumor load, Bone marrow biopsy

## Abstract

In multiple myeloma and its precursor stages, precise quantification of tumor load is of high importance for diagnosis, risk assessment, and therapy response evaluation. Both whole-body MRI, which allows to investigate the complete bone marrow of a patient, and bone marrow biopsy, which is commonly used to assess the histologic and genetic status, are relevant methods for tumor load assessment in multiple myeloma. We report on a series of striking mismatches between the plasma cell infiltration estimating the tumor load from unguided biopsies of the bone marrow at the posterior iliac crest and the tumor load assessment from whole-body MRI.

## Introduction

The current recommendation by the International Myeloma Working Group (IMWG) for the diagnosis of multiple myeloma (MM) requires at least 10% plasma cell infiltration (PCI) in the bone marrow or a biopsy-proven plasmacytoma and at least one myeloma-defining event. These myeloma-defining events determined by the IMWG can be summoned by the SLiM-CRAB acronym (**S**ixty percent or higher PCI, **Li**ght-chain ratio ≥100 of the involved serum light chain vs. the uninvolved serum light-chain, >1 focal lesion ≥5 mm in **M**RI, elevation of **C**alcium concentration in serum by ≥0.25 mmol/l above the upper limit or ≥2.75 mmol/l, respectively, **R**enal insufficiency (creatine clearance <40 ml/min or serum Creatinine >173 mmol/l), **A**nemia with hemoglobin concentration reduced by >2 g/dl below the lower limit or <10 g/dl and ≥1 osteolytic **B**one lesion in CT, PET-CT or skeletal radiography). The guideline by the IMWG for diagnosis of smoldering multiple myeloma (SMM) requires the absence of any myeloma-defining events or amyloidosis and the presence of elevated levels of serum monoclonal protein ≥30 g/L or urinary monoclonal protein ≥500 mg/24 h and/or a PCI of the bone marrow of 10–60% [1]. Because the accurate diagnosis of MM is critical, at least one cross-sectional imaging examination (PET-CT, low-dose whole-body CT, or MRI of the whole body or spine) is recommended for patients before concluding that a patient has SMM or solitary plasmacytoma. The choice between various imaging methods can differ depending on the clinical situation and availability [1, 2]. However, low-dose whole-body CT (wb-CT) was determined to be first-line standard for diagnosing suspected (S)MM by the IMWG and MRI, preferably wb-MRI, is used secondly when findings of wb-CT are negative or inconclusive [3]. At our institution, in absence of contraindications, both wb-MRI and wb-CT are performed when (S)MM is suspected or performed as baseline at initial diagnosis.

In 2005, the IMWG introduced the International Staging System (ISS), which is the most commonly used staging system today and classifies MM patients into three prognostic groups by survival probability (ISS I-III) depending on the serum levels of ß2-microglobulin and serum albumin [4]. The ISS was revised (R-ISS I-III) in 2015 and complemented by the LDH levels (R-ISS I+II: LDH levels not elevated, R-ISS III: LDH levels elevated) and cytogenetic mutation status (R-ISS I+II: no high-risk mutation, R-ISS III: high-risk mutation del(17p) and/or t(4;14) and/or t(14;16)) [5].

For SMM, as defined by the IMWG in 2014, the IMWG undertook a risk stratification that categorized patients into three or, with the addition of prognostically unfavorable cytogenetic aberrations, four subgroups depending on the 2-year risk of progression primarily based on the fulfillment of three criteria: monoclonal protein >2 g/dl, PCI >20%, and the ratio of involved versus noninvolved serum free light chains (ratio >20). Cytogenetic risk factors (t(4;14), t(14;16), +1q, and/or del13q/monosomy 13) have been recognized as an additional fourth criterion [6].

Evaluating tumor load in monoclonal plasma cell disorders is therefore not only essential for diagnosis [1], but also important for individual risk assessment [6] and furthermore for the evaluation of therapy response [7]. Tumor load assessment by bone marrow biopsy is usually based on a single sample from a random site at the iliac crest. A significant downside of biopsies is that these tissue samples are prone to be non-representative as MM is known for its heterogenous tumor load distribution with focal lesions representing accumulated plasma cells in between fewer or even unaffected bone marrow [8]. Given that the presence of ≥60% PCI now is a myeloma-defining event [1], and that a PCI of >20% now contributes to the individual risk assessment in SMM [6], bone marrow biopsies are of high clinical relevance for diagnosis and risk stratification.

Wb-MRI allows to assess the complete bone marrow of the patient including focal lesions [9] and different levels of diffuse PCI [8]. Latifoltojar and colleagues have recently reported that results from bone marrow biopsies can lead to both under- and overestimation of tumor load when compared to MRI [10]. Both bone marrow trephine (BMT) and bone marrow aspirate (BMA) can be used to assess the PCI using multiple analytical methods [11]. However, it has been shown that BMT leads to markedly higher PCI values than BMA [12], with the higher value of both procedures being recommended for diagnosis by the IMWG [1]. Also, biopsy has a higher availability compared to MRI, which has an approximate waiting period of one week at our institution. Especially when situations remain clinically unclear with no serum monoclonal protein determined, histologic assessment via bone marrow biopsy prior to dedicated MRI protocols for MM may support the suspicion of monoclonal plasma cell disorder at an early stage during diagnostic procedure.

In this report, we present four cases, which have been diagnosed as referenced, with striking mismatches between tumor load estimated by PCI from bone marrow biopsy and the appearance of the bone marrow in wb-MRI as well as one case with coincidental biopsy of a small focal lesion showing a great difference in PCI results when comparing BMT versus BMA analysis.

## Case report

### Case 1

**Fig. 1:**
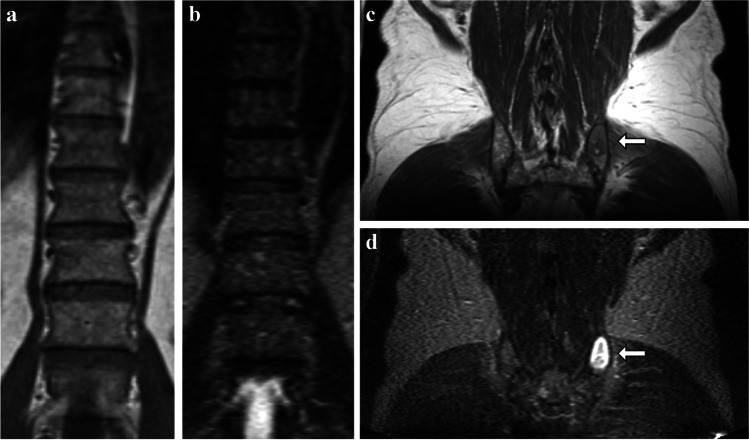
**a–d** Case 1. A 62-year-old male with smoldering multiple myeloma after coincidental biopsy of a large focal lesion. Coronal T1tse (**a**, **c**) and STIR (**b**, **d**) MR images show low to intermediate diffuse infiltration in spine (**a**, **b**) and pelvis (**c**, **d**). However, a biopsy-based PCI of 90% was reported for the bone marrow. This wb-MRI reveals that a focal lesion at the left posterior iliac bone was coincidentally hit in the biopsy (**c**, **d**, white arrows)

A 62-year-old male presented with one criterion met for the diagnosis of MM: bone marrow biopsy performed 16 days after wb-MRI resulted in a 90% PCI in both BMT and BMA. The wb-MRI showed a low to intermediate diffuse infiltration, which was not expected in a patient with a 90% PCI. Additionally, the wb-MRI revealed a 3.0 cm by 1.9 cm large focal lesion at the left posterior iliac crest, which has been hit coincidentally in the biopsy (Fig. 1). As this patient was seen before the introduction of the SLiM-CRAB criteria in 2014 by the IMWG, this patient was initially diagnosed with SMM because of a PCI greater than 10%, as a PCI ≥60% was not yet labeled a myeloma-defining event, and a serum monoclonal protein of 40.9 g/L. [1, 13]. However, this case is a striking example of how a biopsy result of 90% PCI would have led to upstaging to MM and therapy indication under current guidelines caused by an unrepresentative tissue sampling.

### Case 2

**Fig. 2:**
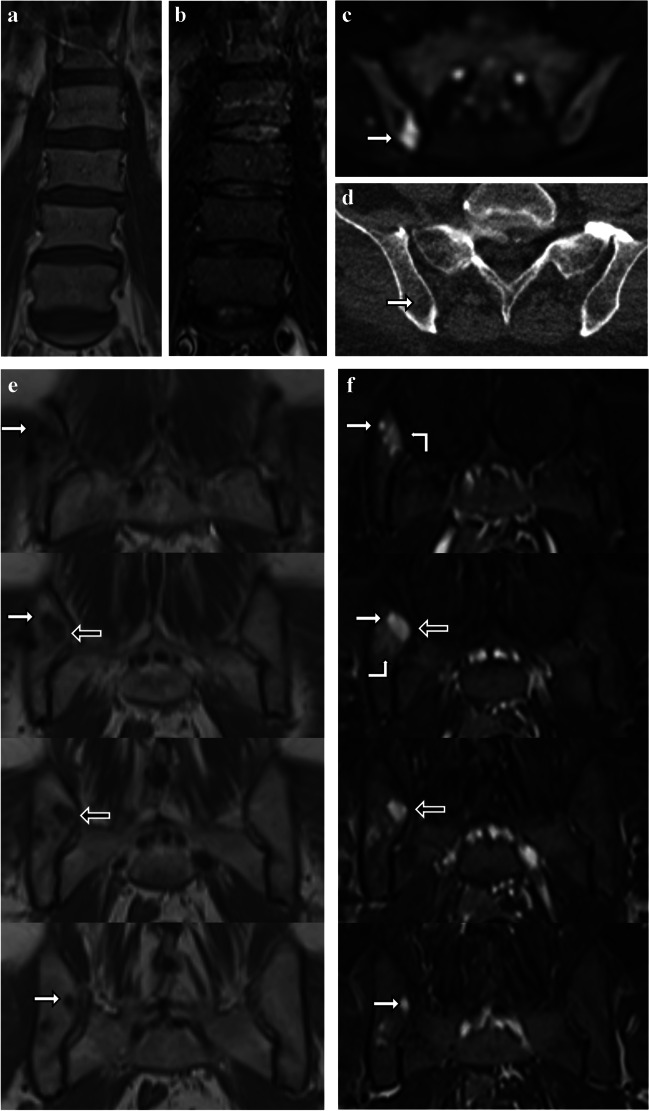
**a–f** Case 2. A 62-year-old male with multiple myeloma after coincidental biopsy of a small focal lesion. **a**, **b** Coronal T1tse (**a**) and STIR (**b**) MR images show physiologic signal of the bone marrow in the spine and pelvis. **c**, **d** The focal lesion shows an increased signal in b800 diffusion weighed imaging (**c**) and an ill-defined, trabecular rarefication in CT imaging (**d**). **e**, **f** Four consecutive coronal T1tse (**e**) and STIR (**f**) MR images from dorsal (upper MR images) to ventral show a small circular area (white arrows) with marked T1-hypointense and marked STIR-hyperintense signal corresponding to the biopsy channel. The biopsy channel (black arrows) is passing through a spherical T1-hypointense and STIR-hyperintense focal lesion of 1.2 cm by 0.8 cm. A diffuse, ill-defined, moderate STIR-hyperintensity, which is most likely corresponding to a bone marrow edema caused by biopsy and aspiration, is located next to the focal lesion and the biopsy channel (**f**, right angle arrows)

A 62-year-old male presented with two criteria met for the diagnosis of MM: a serum free light-chain-ratio (SFLC-r) of 152 with an absolute level of lambda light-chain of 442 mg/l and a PCI of 60% in BMT and 56% in the cytological analysis of BMA from an unguided biopsy of the right posterior iliac crest. In contrast to the bone marrow biopsy results, the wb-MRI performed 7 days after biopsy showed homogenous T1-hyperintense and STIR-hypointense bone marrow in all parts of the examined skeleton with only one 1.2 cm by 0.8 cm focal lesion in the right posterior iliac crest that had been coincidentally hit by the unguided biopsy (Fig. 2).

### Case 3

**Fig. 3:**
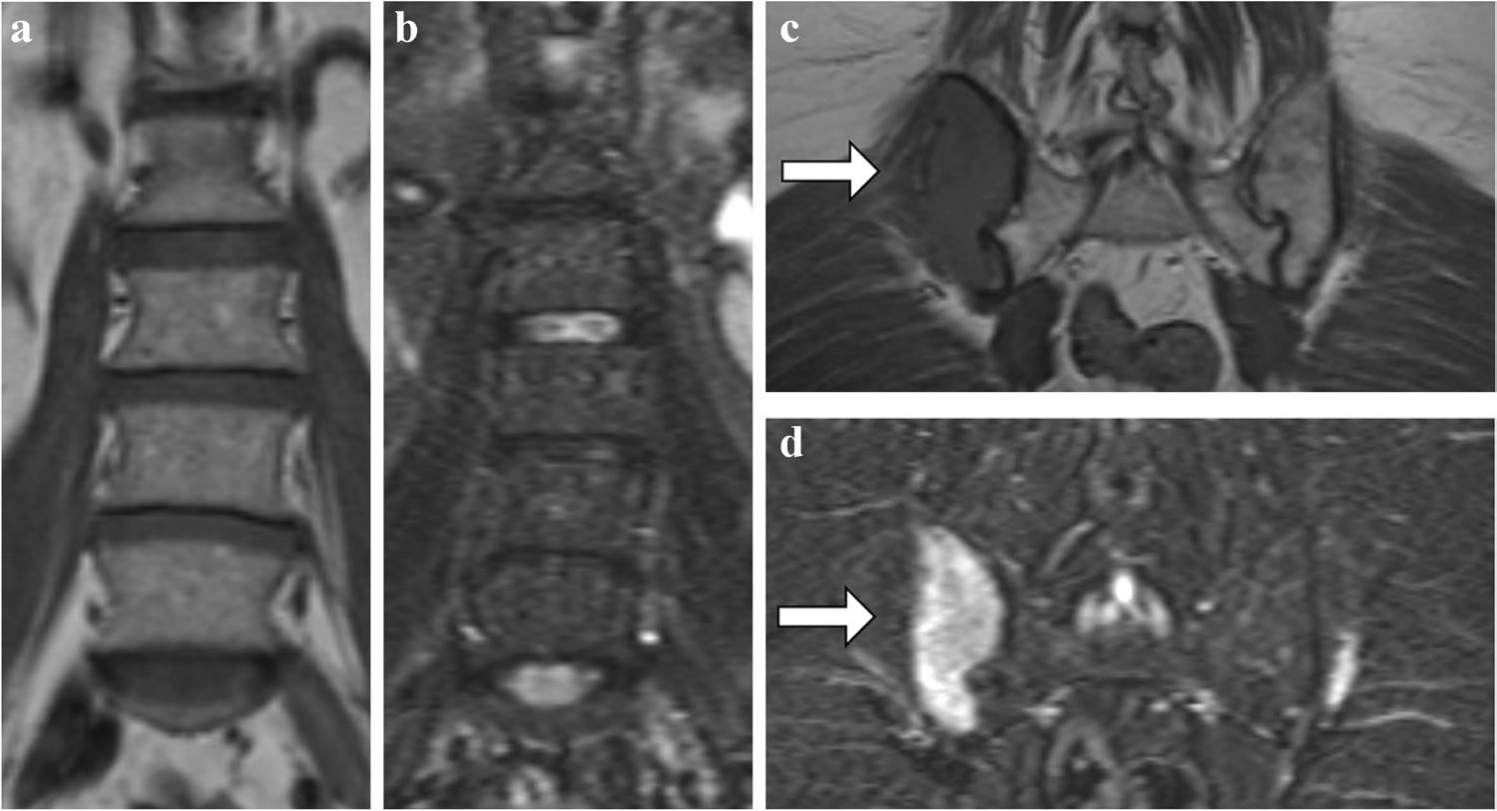
**a–d** Case 3. A 60-year-old female with multiple myeloma with 5% PCI after biopsy of mostly unaffected bone marrow in the posterior iliac crest contralateral to a large focal lesion. Coronal T1tse (**a**, **c**) and STIR (**b**, **d**) MR images show no diffuse infiltration in spine (**a**, **b**) or the left pelvis (**c**, **d**). Wb-MRI revealed a 6.8 cm by 3.0 cm large focal lesion at the right posterior iliac crest (**c**, **d**, white arrows), which would have led to a significantly higher PCI assessment if the right side instead of the left side would have been chosen randomly for biopsy

A 60-year-old female presented with two criteria met for the diagnosis of MM: wb-MRI showed multiple focal lesions, among them a 6.8 cm by 3.0 cm focal lesion at the right posterior iliac crest (Fig. 3). Additionally, wb-CT revealed multiple osteolytic lesions. In contrast to these fulfilled criteria, a bone marrow biopsy 15 weeks earlier at the left posterior iliac crest revealed 5% PCI in both BMT and BMA. It has to be assumed that a biopsy on the opposite side of the pelvis would have led to a markedly higher estimation of tumor burden, as an increased PCI has been reported when targeting focal lesions specifically in CT-guided biopsies compared to unguided biopsies [14].

### Case 4

**Fig. 4:**
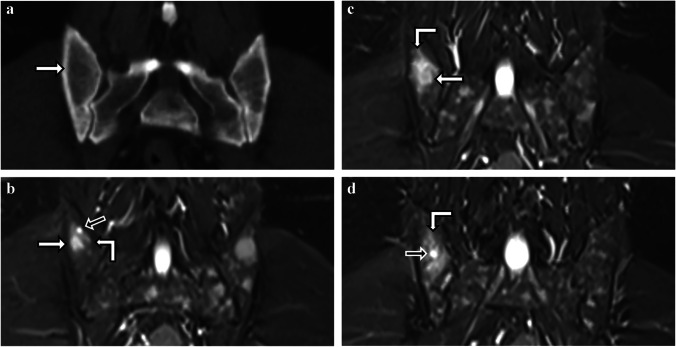
**a–d** Case 4. A 51-year-old male with multiple myeloma presenting with a marked discrepancy between bone marrow trephine and bone marrow aspirate results. **a** Coronal CT image of a scan performed one month prior to the wb-MRI scan showing a 1.0 cm by 0.7 cm osteolytic lesion in the right posterior iliac bone, which is the typical location for an unguided bone marrow biopsy (white arrows). **b-d** Three consecutive coronal STIR MR images from posterior to anterior of the wb-MRI show a small circular area with increased signal in STIR corresponding to the biopsy channel (**b**, **d**, black arrows) and a 1.1 cm by 0.9 cm focal lesion with a STIR-hyperintense signal in the corresponding location to the osteolytic lesion (**b**, **c**, white arrow). Bone marrow edema with intermediate STIR-hyperintensity surrounding the biopsy channel can be detected (right angle arrow)

A 51-year-old male presented with three fulfilled criteria for the diagnosis of MM: with wb-MRI showing multiple focal lesions and wb-CT revealing osteolytic lesions both imaging modalities reported positive findings for the diagnosis of MM. Furthermore, bone marrow biopsy results differed greatly with 60% PCI from BMT, which also met the third criterion for diagnosis, and 13% PCI in the cytological assessment from BMA (Fig. 4). When reading the MRI scan performed two weeks after biopsy, it occurred that the biopsy needle was coincidentally placed in a small osteolytic focal lesion. We speculate that in coincidental biopsies of small focal lesions, BMT still captures the local plasma cell manifestation within the small-sized lesion, whereas a potential dilution effect during the extraction of BMA could lead to lower PCI results in the cytological assessment [15], causing the disparity between histologically and cytologically estimated PCI [12].

## Discussion

According to the current guidelines, biopsy results contribute to the diagnosis of plasma cell diseases and influence the decision to start systemic therapy, as ≥60% PCI is now a myeloma-defining event [1]. Moreover, biopsy results are important for individual risk assessment in SMM [6, 16] as well as for response and minimal residual disease assessment [7]. However, given the heterogenous tumor load distribution [10] and spatial genomic heterogeneity [17] of MM, it is of utmost importance in both research and clinical setting to be aware of the fact from which MRI-phenotypic tissue — a focal lesion or a homogenous area of diffuse infiltration — the specimen was derived, and whether the results from the biopsy are representative for the complete bone marrow of the patient, or if they may only represent a locally confined structure.

Imaging prior to biopsy allows to identify locations for sampling representative tissue for PCI calculation, or to identify locations with specific MRI-phenotypes as for example large focal lesions, which were associated with adverse outcome [18], corresponding bone destruction [19] and contained biologically advanced tumor cells [17], and can be sampled via image-guided biopsy [20]. In cases where no imaging was performed prior to biopsy and a PCI ≥60% is the only myeloma-defining event indicating systemic treatment, wb-MRI should be performed to assure that the PCI is representative for the overall tumor burden and not caused by a coincidental hit of a focal lesion. Wb-MRI might also bring additional benefit in a reverse scenario, in which the PCI is less than 60% and lesions might have been missed. Of note, it has recently been demonstrated that machine learning algorithms can predict a surrogate parameter for plasma cell infiltration from MRI [21], which might be of help to overcome shortcomings of biopsies, such as their invasiveness and the possibility of sampling of none-representative tissue, as shown in this case series.

We conclude that tumor load assessment should not be performed by biopsies alone but accompanied by MRI, preferably of the whole body [22–24], in order to assure that the sampled tissue for histological or genetic analysis is either representative for the whole body, or intendedly obtained from a targeted focal lesion to assess the most advanced biological alterations of malignant plasma cells.
